# Concurrent Diabetic Muscle Infarction and JAK2 Exon 12-Positive Polycythemia Vera: A Rare Dual Diagnosis

**DOI:** 10.7759/cureus.115831

**Published:** 2026-09-05

**Authors:** Isabel V Rodrigues, Costantin Sitari, Rosário Blanco Saez, Joao Santos, Paula Nogueira, Ana Pimenta de Castro

**Affiliations:** 1 Internal Medicine, Unidade Local de Saúde do Algarve, Faro, PRT

**Keywords:** diabetes mellitus, diabetic muscle infarction, erythrocytosis, jak2 exon 12, myeloproliferative neoplasm, polycythemia vera

## Abstract

Diabetic muscle infarction (DMI) is an uncommon and frequently underrecognized microvascular complication of longstanding diabetes mellitus characterized by spontaneous ischemic necrosis of skeletal muscle. Polycythemia vera (PV) associated with JAK2 exon 12 mutations represents a rare subtype of myeloproliferative neoplasm that typically presents with isolated erythrocytosis and suppressed serum erythropoietin levels. The coexistence of these two uncommon disorders is exceptionally rare and may create significant diagnostic challenges because painful limb swelling can mimic thrombotic, infectious, inflammatory, or neoplastic conditions.

We report the case of a 52-year-old man with poorly controlled type 2 diabetes mellitus who presented with a 3-week history of progressive atraumatic pain and swelling of the left thigh. Doppler ultrasonography excluded deep vein thrombosis and abscess formation, while magnetic resonance imaging demonstrated diffuse muscle edema, short tau inversion recovery (STIR) hyperintensity, and characteristic findings consistent with DMI. Laboratory investigations revealed marked erythrocytosis (hemoglobin 19.9 g/dL), thrombocytosis, and suppressed serum erythropoietin levels. Bone marrow examination demonstrated hypercellularity with erythroid predominance, and molecular testing identified a JAK2 exon 12 mutation, fulfilling contemporary diagnostic criteria for polycythemia vera. Conservative management of DMI together with therapeutic phlebotomy for PV resulted in progressive clinical improvement.

This case highlights the importance of maintaining a broad differential diagnosis when evaluating painful limb swelling in patients with diabetes mellitus and persistent erythrocytosis. Recognition of coexisting disorders through careful integration of clinical assessment, imaging, and hematological investigation allows timely diagnosis, avoids unnecessary invasive procedures, and facilitates appropriate disease-specific management.

## Introduction

Diabetic muscle infarction (DMI), also known as diabetic myonecrosis, is a rare complication of longstanding diabetes mellitus resulting from spontaneous ischemic necrosis of skeletal muscle. Its pathogenesis is thought to involve diabetes-related microangiopathy and endothelial dysfunction, resulting in impaired skeletal muscle perfusion, tissue ischemia, and ultimately muscle necrosis. It predominantly affects patients with poorly controlled diabetes and advanced microvascular disease and most commonly involves the thigh or calf musculature. Although uncommon, DMI should be considered in diabetic patients presenting with acute or subacute atraumatic limb pain and swelling because delayed recognition frequently leads to unnecessary invasive investigations or surgical intervention. Magnetic resonance imaging (MRI) is regarded as the diagnostic modality of choice owing to its characteristic imaging features and ability to exclude alternative diagnoses. Conservative treatment with rest, analgesia, and optimization of glycemic control generally results in favorable outcomes, although recurrence is not uncommon [1-6].

The differential diagnosis of painful muscle swelling is broad and includes deep vein thrombosis, infectious myositis, inflammatory myopathies, soft tissue sarcoma, compartment syndrome, and DMI. Clinical presentation alone is often insufficient to distinguish among these entities, making careful interpretation of imaging findings essential for establishing the correct diagnosis. Muscle biopsy should generally be avoided when clinical and radiological findings are typical because it may delay recovery and increase the risk of complications [1-6].

Polycythemia vera (PV) is a chronic myeloproliferative neoplasm characterized by autonomous erythrocytosis resulting from constitutive activation of the JAK-STAT signaling pathway, most commonly due to JAK2 mutations. Whereas the more common JAK2 V617F-positive form of PV frequently presents with erythrocytosis accompanied by leukocytosis and thrombocytosis, JAK2 exon 12-positive disease is less common and typically manifests as isolated erythrocytosis with suppressed serum erythropoietin concentrations and characteristic bone marrow morphology. Because PV is associated with hyperviscosity and an increased risk of arterial and venous thrombosis, painful limb swelling may initially be interpreted as a thrombotic manifestation rather than an independent musculoskeletal disorder. Current diagnostic criteria integrate persistent erythrocytosis, low serum erythropoietin levels, characteristic bone marrow findings, and demonstration of a pathogenic JAK2 mutation [7,8].

The coexistence of DMI and polycythemia vera is exceptionally uncommon and presents a unique diagnostic challenge. In such patients, muscle swelling may be incorrectly attributed to the thrombotic complications of PV, whereas persistent erythrocytosis may be dismissed as a reactive phenomenon associated with acute inflammation. This case illustrates the importance of maintaining a broad differential diagnosis and integrating clinical, radiological, and hematological findings to recognize two distinct disorders requiring different therapeutic approaches.

## Case presentation

A 52-year-old man of Cape Verdean origin, residing in Portugal for more than 30 years, presented to the Emergency Department with a 3-week history of progressively worsening atraumatic pain and swelling involving the anterior aspect of the left thigh. The pain had progressively increased in intensity, limiting ambulation and daily activities. He denied recent trauma, strenuous physical exercise, fever, constitutional symptoms, or recent travel.

His medical history was significant for poorly controlled type 2 diabetes mellitus, diagnosed in 2011, for which he had remained untreated and without regular medical follow-up until January 2020. Additional comorbidities included recently diagnosed arterial hypertension and untreated dyslipidemia. Fundoscopic examination and urine albumin-to-creatinine ratio measurement were not performed during this admission, and the available medical records contained no reliable documentation of pre-existing diabetic retinopathy, nephropathy, or peripheral neuropathy. Therefore, the presence and extent of diabetic microvascular end-organ damage could not be retrospectively established.

Initial treatment prescribed by his primary care physician, consisting of nonsteroidal anti-inflammatory drugs and simple analgesics, failed to provide symptomatic relief, prompting hospital referral.

On admission, the patient was afebrile and hemodynamically stable. Physical examination demonstrated a firm, tender, indurated swelling involving the distal two-thirds of the anterior compartment of the left thigh, with increased local temperature but without erythema, fluctuance, or skin ulceration. Distal pulses were palpable and symmetrical, and there were no neurological deficits or evidence of compartment syndrome. No peripheral lymphadenopathy or hepatosplenomegaly was detected.

Initial laboratory investigations revealed marked erythrocytosis (hemoglobin 19.9 g/dL; hematocrit 62%), thrombocytosis, mild leukocytosis, elevated inflammatory markers, and poor glycemic control. Serum erythropoietin levels were suppressed, raising suspicion of an underlying myeloproliferative neoplasm. Renal function remained preserved despite severe hyperglycemia. Serum creatine kinase and urine myoglobin levels were not measured during admission. Laboratory findings are summarized in Table 1.

**Table 1:** Laboratory investigations at admission The results demonstrate marked erythrocytosis and thrombocytosis associated with suppressed erythropoietin levels, together with elevated inflammatory markers and poor glycemic control. Renal function was preserved despite significant hyperglycemia.

Parameter	Result	Reference range	Interpretation
Hemoglobin	19.9 g/dL	13.0–17.0 g/dL	Absolute erythrocytosis
Hematocrit	0.62 L/L	0.40–0.50 L/L	Elevated
Platelets	618 × 10³/μL	150–400 × 10³/μL	Thrombocytosis
Leukocytes	11.4 × 10³/μL	4.0–10.0 × 10³/μL	Mild leukocytosis
C-reactive protein (CRP)	66 mg/L	<5 mg/L	Elevated
Sodium	131 mmol/L	136–144 mmol/L	Hyponatremia
Potassium	5.4 mmol/L	3.3–5.1 mmol/L	Hyperkalemia
Serum creatinine	0.9 mg/dL	0.7–1.3 mg/dL	Normal
Blood urea nitrogen (BUN)	27 mg/dL	8.4–25.7 mg/dL	Slightly elevated
HbA1c (January 2020)	13.6%	4.8–5.9%	Poor glycemic control
Fasting glucose	342 mg/dL	70–99 mg/dL	Hyperglycemia
D-dimers	558 ng/mL	<500 ng/mL	Slightly elevated

Imaging evaluation was undertaken to characterize the lesion and to exclude alternative causes of painful limb swelling, including deep vein thrombosis, infectious myositis, soft-tissue neoplasms, and inflammatory muscle disease. Compression Doppler ultrasonography of the left lower limb excluded deep vein thrombosis and demonstrated diffuse enlargement and increased echogenicity of the vastus lateralis muscle without abscess formation or focal fluid collection (Figure 1).

**Figure 1:**
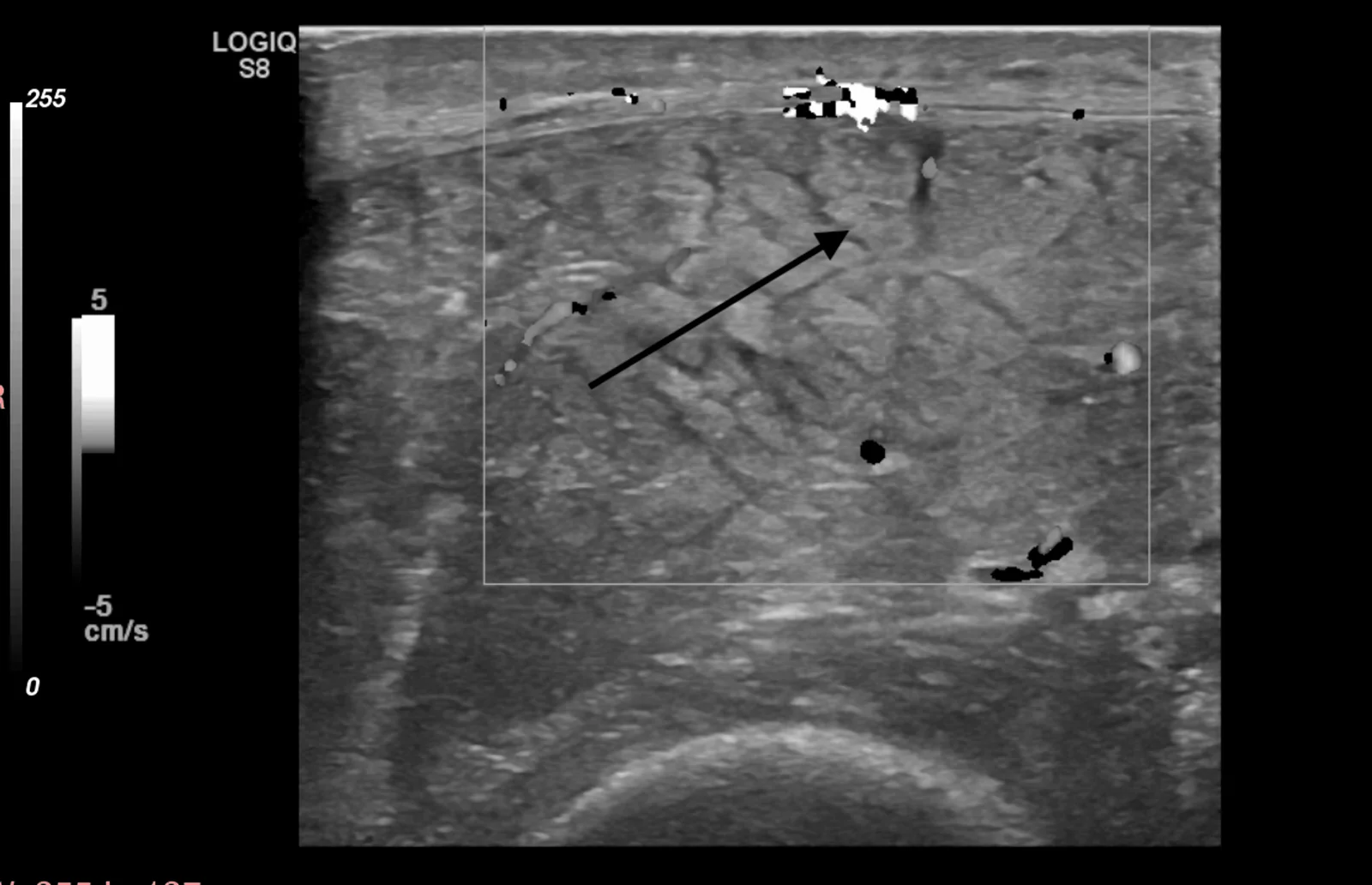
Grayscale ultrasonography of the left thigh Grayscale ultrasonography of the left thigh demonstrating diffuse enlargement and heterogeneous echotexture of the vastus lateralis muscle (arrow), without focal fluid collection or sonographic evidence of abscess formation.

Because symptoms persisted and ultrasonographic findings were nonspecific, magnetic resonance imaging (MRI) of the lower limbs was subsequently performed. MRI of the left thigh demonstrated diffuse enlargement of the vastus lateralis muscle, with extensive intramuscular edema and hyperintense signal on T2-weighted fat-suppressed and STIR sequences (Figures 2, 3). Post-contrast T1-weighted fat-suppressed images showed diffuse heterogeneous muscular enhancement with associated perifascial edema (Figures 4, 5). No focal abscess, soft tissue mass, osteomyelitis, or imaging features suggestive of inflammatory myopathy were identified. These findings were considered characteristic of DMI.

**Figure 2:**
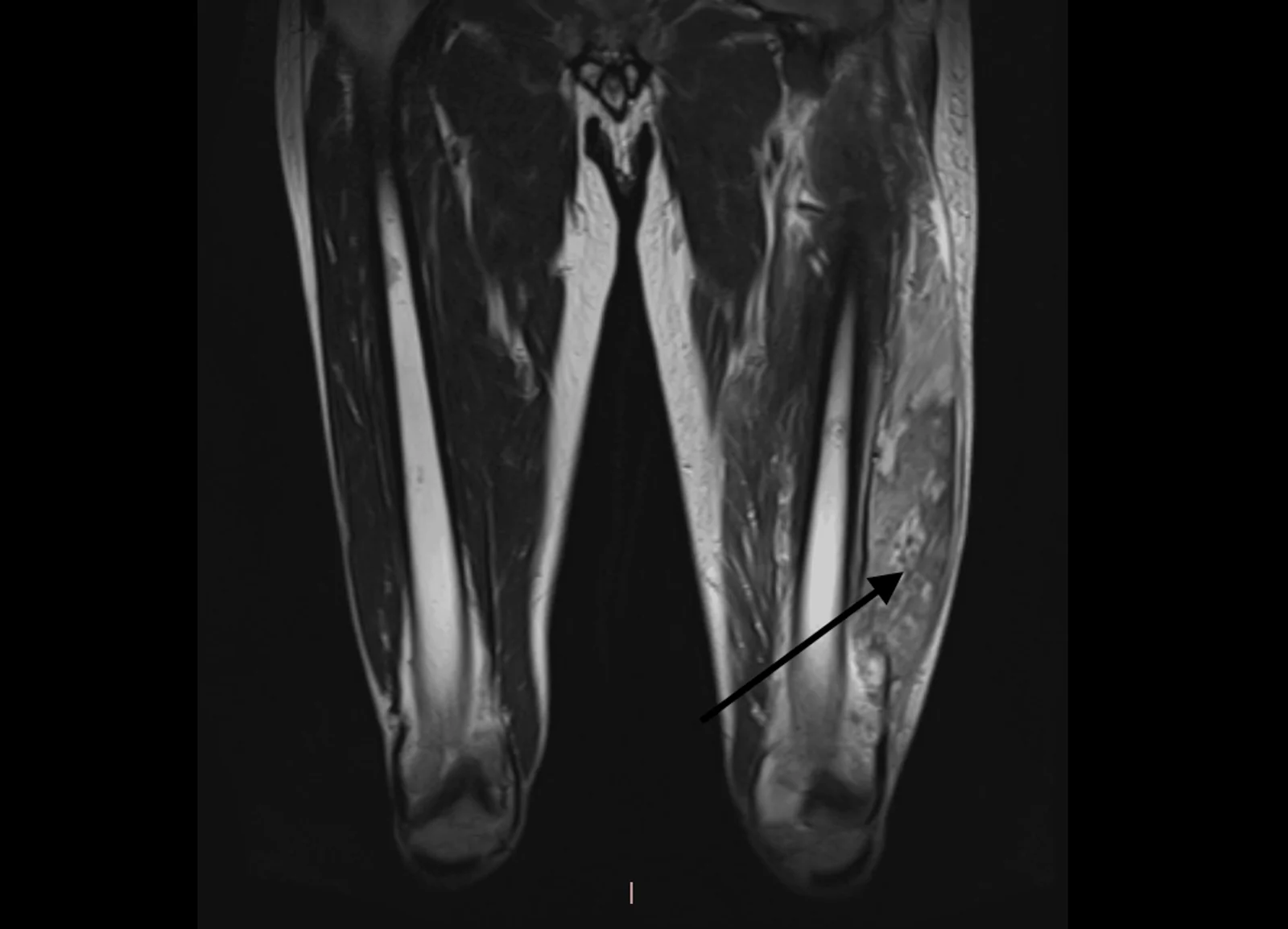
Coronal T2 magnetic resonance image Coronal T2-weighted fat-suppressed magnetic resonance image demonstrating diffuse enlargement of the left vastus lateralis muscle with extensive intramuscular hyperintensity (arrow), consistent with diffuse muscle edema characteristic of diabetic muscle infarction.

**Figure 3:**
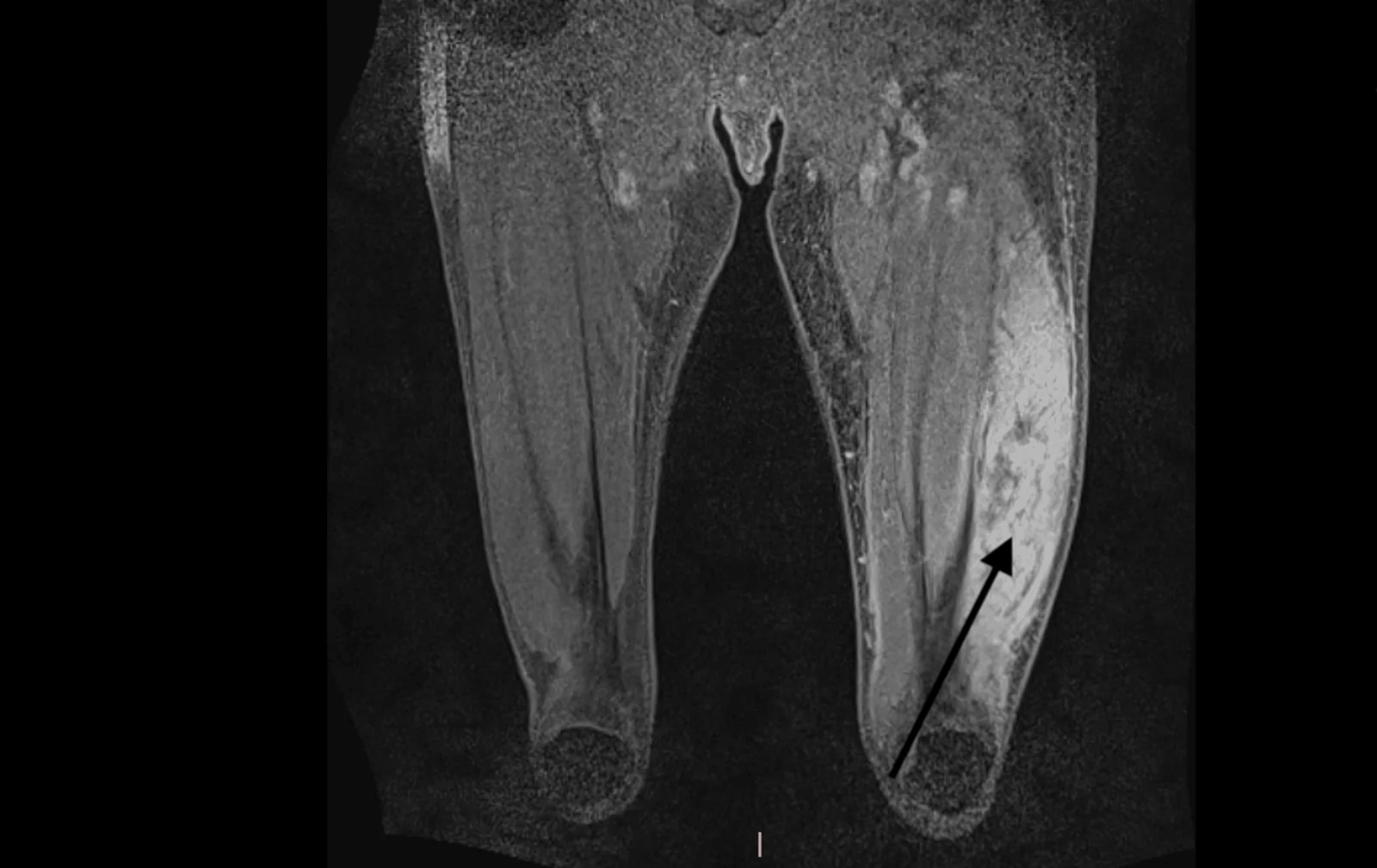
Coronal STIR magnetic resonance image Coronal STIR magnetic resonance image demonstrating extensive hyperintense signal throughout the left vastus lateralis muscle (arrow), reflecting diffuse intramuscular edema and illustrating the longitudinal extent of diabetic muscle infarction. STIR: short tau inversion recovery

**Figure 4:**
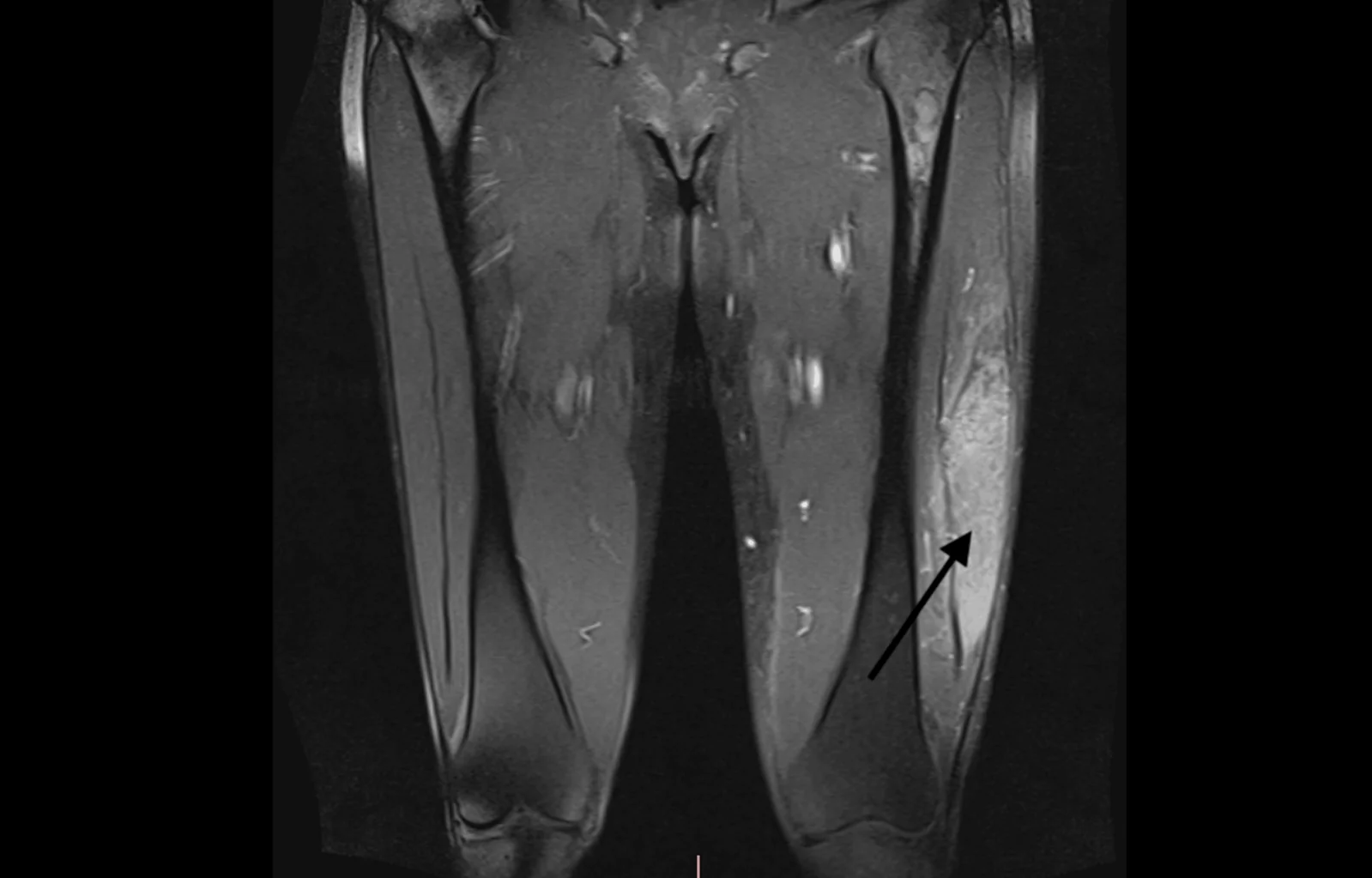
Coronal T1 post-contrast magnetic resonance image Coronal T1-weighted fat-suppressed post-contrast magnetic resonance image demonstrating diffuse heterogeneous enhancement of the left vastus lateralis muscle (arrow), with associated perifascial edema and no evidence of focal abscess or osteomyelitis.

**Figure 5:**
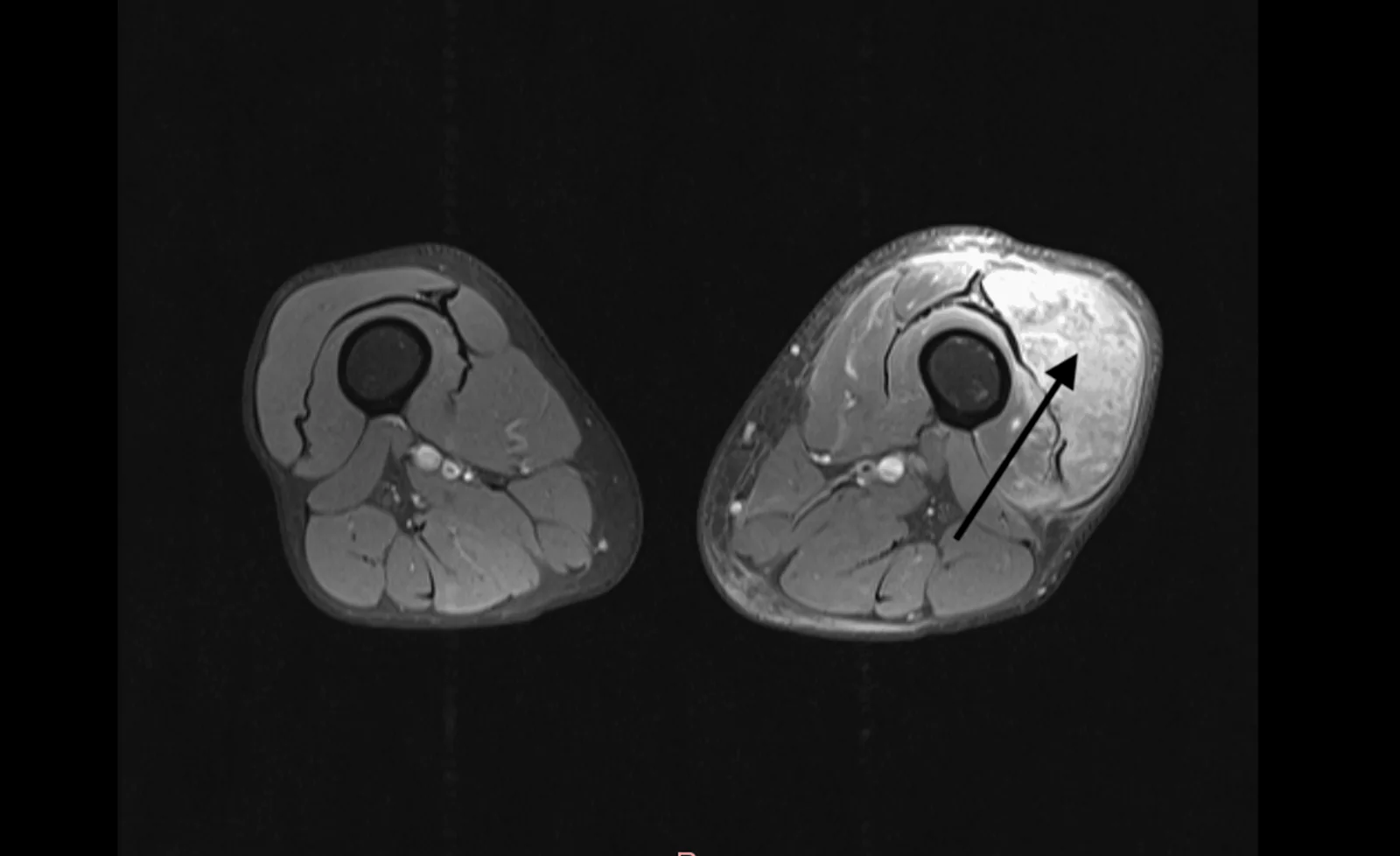
Axial T1 post-contrast magnetic resonance image Axial T1-weighted fat-suppressed post-contrast magnetic resonance image showing diffuse enlargement and heterogeneous enhancement of the left vastus lateralis muscle (arrow), with surrounding fascial edema and no evidence of focal fluid collection or soft tissue mass.

The principal imaging findings are summarized in Table 2.

**Table 2:** Imaging findings of the left thigh and lower limbs Doppler ultrasonography excluded deep vein thrombosis and abscess formation, whereas magnetic resonance imaging demonstrated characteristic features of diabetic muscle infarction, allowing a confident diagnosis without the need for muscle biopsy.

Imaging modality	Findings	Interpretation	Figures
Ultrasound (left thigh)	Diffuse enlargement and increased echogenicity of the vastus lateralis without abscess or focal fluid collection	Diffuse muscle enlargement without abscess or deep vein thrombosis	Figure 1
MRI	Diffuse enlargement of the vastus lateralis muscle with extensive intramuscular edema and hyperintense signal on T2-weighted fat-suppressed and STIR sequences; diffuse heterogeneous muscular enhancement and perifascial edema on post-contrast T1-weighted fat-suppressed images; no focal abscess or soft tissue mass.	Characteristic of diabetic muscle infarction	Figures 2–5

Hematological evaluation

Although DMI explained the patient's musculoskeletal symptoms, the marked erythrocytosis and thrombocytosis were considered disproportionate to the inflammatory response and prompted further hematological investigation.

Serum erythropoietin concentration was suppressed. Bone marrow biopsy demonstrated hypercellularity with erythroid predominance, and molecular analysis identified a JAK2 exon 12 mutation, while testing for BCR-ABL1, CALR, and MPL mutations was negative. Collectively, these findings fulfilled current diagnostic criteria for polycythemia vera.

Differential diagnosis

The patient's presentation with atraumatic thigh pain and swelling prompted consideration of several differential diagnoses, including deep vein thrombosis, infectious myositis, pyomyositis, inflammatory myopathy, soft tissue sarcoma, and DMI.

Deep vein thrombosis was excluded by compression Doppler ultrasonography, which demonstrated patent deep venous structures without evidence of thrombosis. Infectious myositis and pyomyositis were considered unlikely because the patient was afebrile, blood cultures remained negative, and MRI demonstrated no abscess formation or peripheral contrast enhancement. Inflammatory myopathy was also considered; however, the focal nature of the lesion, absence of generalized muscle weakness, and characteristic MRI findings argued strongly against this diagnosis. Likewise, the absence of a focal soft tissue mass or bone involvement excluded a neoplastic process.

Taken together, the patient's longstanding poorly controlled diabetes mellitus, typical clinical presentation, and characteristic MRI findings strongly supported the diagnosis of DMI.

Table 3 summarizes the principal differential diagnoses considered during the diagnostic evaluation.

**Table 3:** Differential diagnoses considered for muscle swelling in a diabetic patient This table outlines the main differential diagnoses considered in the evaluation of unexplained muscle swelling in a patient with diabetes mellitus. Infectious myositis and abscess were considered unlikely due to the absence of systemic infection and fluid collections. Neoplastic processes were excluded by the lack of a focal mass on MRI. Inflammatory myopathies were less likely due to the focal nature of symptoms and absence of muscle weakness or enzyme elevation. The overall findings strongly supported diabetic muscle infarction. The absence of systemic infection signs, lack of abscess on imaging, and the presence of typical MRI features, combined with a history of poorly controlled diabetes, favored the diagnosis of diabetic muscle infarction.

Differential diagnosis	Reason for exclusion/consideration
Deep vein thrombosis	Excluded by Doppler ultrasonography
Infectious myositis/abscess	No fever or systemic infection; MRI showed no abscess or pyomyositis
Inflammatory myopathy	Focal rather than symmetrical involvement; imaging not compatible with inflammatory myopathy
Soft tissue sarcoma	No focal soft-tissue mass or neoplastic features on MRI
Diabetic muscle infarction	Typical clinical presentation in poorly controlled diabetes with characteristic MRI findings

Hospital course and treatment

The patient was admitted to the Internal Medicine department, with close collaboration with Orthopedic Surgery and Hematology.

Supportive treatment for DMI consisted of bed rest during the acute phase, progressive mobilization as tolerated, multimodal analgesia with metamizole, tramadol, and paracetamol, and optimization of glycemic control using basal insulin therapy combined with oral antidiabetic medication. Muscle biopsy was not performed because the clinical presentation and MRI findings were considered diagnostic, and biopsy has been associated with delayed recovery and an increased risk of complications in patients with DMI.

Serum erythropoietin concentration was suppressed. Bone marrow biopsy demonstrated hypercellularity with erythroid predominance, and molecular analysis identified a JAK2 exon 12 mutation, while testing for BCR-ABL1, CALR, and MPL mutations was negative. These findings fulfilled current diagnostic criteria for polycythemia vera.

Following confirmation of the diagnosis, therapeutic phlebotomy was initiated under Hematology supervision. A total of 6 phlebotomy sessions, each removing approximately 300 mL of blood, were performed during hospitalization, resulting in progressive reduction in hematocrit and improvement in hyperviscosity-related laboratory abnormalities.

The patient's pain gradually improved, and the thigh swelling progressively regressed throughout hospitalization without the need for surgical intervention.

Follow-up and outcome

After 15 days of hospitalization, the patient experienced marked improvement in pain, reduction of thigh swelling, and recovery of functional capacity, allowing independent ambulation. Hyponatremia and hyperkalemia resolved with intravenous fluid therapy and appropriate medical treatment. Glycemic control also improved following initiation of insulin therapy.

The patient was discharged receiving aspirin 100 mg once daily for primary thromboprophylaxis associated with polycythemia vera, together with antihypertensive therapy, basal insulin, and oral antidiabetic medication. Therapeutic phlebotomy was continued in the outpatient setting under Hematology supervision, with a target hematocrit of <45%.

At the available follow-up, the patient remained clinically stable, with no documented recurrence of DMI. Therapeutic phlebotomy was continued under Hematology supervision with a target hematocrit of <45%. However, the exact post-discharge hematocrit values and longer-term follow-up data were not available for inclusion in this report.

## Discussion

DMI, also referred to as diabetic myonecrosis, is a rare complication of longstanding diabetes mellitus that predominantly affects patients with poor glycemic control and advanced microvascular disease. Despite its characteristic clinical and radiological features, DMI remains underrecognized in clinical practice and is frequently mistaken for more common conditions such as deep vein thrombosis, infectious myositis, inflammatory myopathy, or soft tissue neoplasms. Delayed recognition often results in unnecessary invasive investigations, prolonged hospitalization, and inappropriate treatment. Consequently, maintaining a high index of suspicion is essential when evaluating diabetic patients presenting with atraumatic limb pain and swelling. [1-6]

The diagnosis of DMI relies on the integration of clinical presentation with characteristic MRI findings. In our patient, MRI demonstrated diffuse enlargement of the vastus lateralis associated with extensive intramuscular edema and STIR hyperintensity without evidence of abscess, soft-tissue mass, or osteomyelitis. These findings, together with the patient's history of poorly controlled diabetes mellitus, were considered diagnostic of DMI and allowed muscle biopsy to be safely avoided. This approach is supported by current evidence, which recommends reserving biopsy for atypical presentations because of its association with delayed healing, prolonged recovery, and secondary infection [2,4,5].

An important feature of this case was the coexistence of persistent erythrocytosis and thrombocytosis, abnormalities that could not be explained solely by the inflammatory process associated with DMI. Rather than attributing these findings to dehydration or a reactive response, additional hematological investigation was pursued. The diagnosis of polycythemia vera was established according to current diagnostic criteria, integrating absolute erythrocytosis, suppressed serum erythropoietin concentration, characteristic bone marrow morphology, and identification of a JAK2 exon 12 mutation. This illustrates the importance of reassessing unexpected laboratory abnormalities even after an apparently satisfactory explanation has been identified for the presenting complaint. Current international guidelines emphasize that persistent erythrocytosis should always prompt investigation for an underlying myeloproliferative neoplasm, particularly when accompanied by thrombocytosis and low erythropoietin levels [7,8].

Recent evidence has further refined the diagnosis and management of polycythemia vera, emphasizing risk-adapted therapeutic strategies, thrombotic risk stratification, and the integration of clinical, morphological, and molecular findings, as reflected in contemporary expert recommendations and disease classifications [9-12].

The coexistence of DMI and polycythemia vera is exceptionally uncommon. Although both disorders involve ischemic mechanisms, their underlying pathophysiology differs substantially. DMI is believed to result primarily from diabetic microangiopathy, endothelial dysfunction, and microvascular ischemia. In PV, marked erythrocytosis alters blood rheology by increasing viscosity and impairing microvascular blood flow, while platelet activation and thrombocytosis may further promote a prothrombotic state. In this patient, these abnormalities may have constituted a hemodynamic “second hit,” further compromising skeletal muscle perfusion in a vascular bed already vulnerable to diabetes-related microangiopathy. However, a direct causal contribution of PV to the muscle infarction cannot be established from a single case, and this proposed interaction remains biologically plausible but speculative.

This case also highlights an important diagnostic pitfall. In patients with polycythemia vera, painful limb swelling may easily be interpreted as a thrombotic complication, whereas in diabetic patients, elevated inflammatory markers may reinforce the suspicion of infection. Premature diagnostic closure could therefore delay recognition of DMI or overlook an underlying hematological disorder. A systematic diagnostic strategy incorporating clinical assessment, vascular imaging, MRI, and appropriate hematological investigation was essential for establishing both diagnoses while avoiding unnecessary invasive procedures.

From a practical perspective, this case reinforces three important clinical messages. First, DMI should always be considered in patients with poorly controlled diabetes presenting with acute or subacute atraumatic muscle pain and swelling. Second, persistent erythrocytosis and thrombocytosis should never be assumed to represent reactive abnormalities and require investigation for an underlying myeloproliferative neoplasm. Finally, multidisciplinary collaboration between Internal Medicine, Radiology, Orthopedic Surgery, and Hematology was fundamental to achieving an accurate diagnosis and implementing timely disease-specific management.

This case illustrates how persistent diagnostic curiosity and systematic reassessment of unexpected findings can prevent premature diagnostic closure when multiple uncommon conditions coexist.

## Conclusions

Diabetic muscle infarction should be considered in patients with longstanding, poorly controlled diabetes mellitus presenting with acute or subacute atraumatic limb pain and swelling. Early recognition of its characteristic MRI findings allows prompt diagnosis, avoids unnecessary muscle biopsy, and facilitates appropriate conservative management. This case illustrates the importance of investigating persistent erythrocytosis and thrombocytosis rather than attributing these abnormalities solely to the inflammatory response. Comprehensive hematological evaluation led to the diagnosis of JAK2 exon 12-positive polycythemia vera, a distinct condition requiring disease-specific management. Although the coexistence of diabetic muscle infarction and polycythemia vera is exceptionally uncommon, this case emphasizes the importance of maintaining a broad differential diagnosis and avoiding premature diagnostic closure. Careful integration of clinical assessment, imaging findings, laboratory investigations, and multidisciplinary collaboration enabled accurate diagnosis and timely treatment of two independent disorders.

Additional case reports and larger observational studies are needed to better define the clinical significance of this uncommon association and determine whether it has implications for diagnosis, management, or prognosis.
